# Temporal and spatial association of *Streptococcus suis* infection in humans and porcine reproductive and respiratory syndrome outbreaks in pigs in northern Vietnam

**DOI:** 10.1017/S0950268815000990

**Published:** 2015-05-22

**Authors:** V. T. L. HUONG, L. V. THANH, V. D. PHU, D. T. TRINH, K. INUI, N. TUNG, N. T. K. OANH, N. V. TRUNG, N. T. HOA, J. E. BRYANT, P. W. HORBY, N. V. KINH, H. F. L. WERTHEIM

**Affiliations:** 1 Wellcome Trust Major Overseas Programme, Oxford University Clinical Research Unit, Hanoi, Vietnam; 2 Nuffield Department of Clinical Medicine, University of Oxford, Oxford, UK; 3 National Hospital for Tropical Diseases, Hanoi, Vietnam; 4 Food and Agriculture Organization, Hanoi, Vietnam; 5 Epidemiology Division, Department of Animal Health, Hanoi, Vietnam; 6 National Centre of Veterinary Diagnosis, Hanoi, Vietnam; 7 Wellcome Trust Major Overseas Programme, Oxford University Clinical Research Unit, Ho Chi Minh City, Vietnam

**Keywords:** Case-control, pigs, porcine reproductive and respiratory syndrome, secondary infection, *Streptococcus suis*, Vietnam, zoonosis

## Abstract

Porcine reproductive and respiratory syndrome (PRRS) outbreaks in pigs are associated with increased susceptibility of pigs to secondary bacterial infections, including *Streptococcus suis* – an important zoonotic pathogen causing bacterial meningitis in humans. This case-control study examined the association between human *S. suis* infection and PRRS outbreaks in pigs in northern Vietnam. We included 90 *S. suis* case-patients and 183 non-*S. suis* sepsis controls from a referral hospital in Hanoi in 2010, a period of major PRRS epizootics in Vietnam. PRRS exposure was determined using data from the National Centre of Veterinary Diagnosis. By univariate analysis, significantly more *S. suis* patients were reported residing in or adjacent to a PRRS district compared to controls [odds ratio (OR) 2·82, 95% confidence interval (CI) 1·35–5·89 and OR 3·15, 95% CI 1·62–6·15, respectively]. Only residency in adjacent districts remained significantly associated with risk of *S. suis* infection after adjusting for sex, occupation, and eating practices. SaTScan analysis showed a possible cluster of *S. suis* infection in humans around PRRS confirmed locations during the March–August period. The findings indicate an epidemiological association between PRRS in pigs and *S. suis* infections in humans. Effective strategies to strengthen control of PRRS in pigs may help reduce transmission of *S. suis* infection to humans.

## INTRODUCTION

*Streptococcus suis* is a common Gram-positive bacterium in the normal flora of swine respiratory, gastrointestinal and reproductive tract [1]. Particular *S. suis* serotypes are more virulent and can cause severe infections in both pigs and humans [2]. Human infections with *S. suis* are common in Southeast Asia and China. Most patients with *S. suis* infection present with meningitis and sepsis, with a mortality rate of ~13% [3]. Among survivors, hearing loss develops in about 39% of patients, followed by vestibular dysfunction in 23% [3], which may be reduced in those who received early dexamethasone adjunctive to antibiotics [4]. In Vietnam, *S. suis* is the most important pathogen causing bacterial meningitis in adult populations [4, 5]. Significantly, more *S. suis* patients have an occupation related to pigs [odds ratio (OR) 5·52] or a history of eating uncooked or undercooked pig products (OR 4·44) compared to the general population [6]. Small-scale household pig rearing is very common in many parts of Vietnam and accounts for the majority of pork production [7, 8], with slaughter and meat-processing activities typically occuring at unregulated facilities especially in the northern region [9].

Human *S. suis* cases have been suggested to be linked to the occurrence of porcine respiratory and reproductive syndrome (PRRS) virus outbreaks in pigs in northern Vietnam [5]. Major epizootics of PRRS caused devastating losses to the swine sector of Vietnam in 2007–2010 [10]. In 2010, an increase in the number of human *S. suis* cases coincided with PRRS outbreaks in both northern and southern Vietnam, suggesting a possible temporal association [11]. Experimental studies in pigs have demonstrated that *S. suis* infection leads to increased severity of PRRS disease, and that PRRS virus infection increases susceptibility to *S. suis* [12–14]. Consequently, there may be an increased risk of *S. suis* transmission to humans through exposure to pigs concomitantly infected with PRRS virus and *S. suis* bacteria. Sufficient data are not available to confirm or refute this hypothesis. We therefore conducted this study to investigate temporal and spatial associations between human *S. suis* infections and PRRS outbreaks in pigs during a period of major epizootic activity in northern Vietnam in 2010. This report contributes to the evidence base for assessing risk factors for zoonotic transmission of *S. suis* infection.

## METHODS

### Study design

This retrospective case-control study included cases with confirmed *S. suis* infection and hospital controls diagnosed with sepsis (not caused by *S. suis*) at the National Hospital for Tropical Diseases (NHTD), a tertiary care and treatment centre for infectious diseases based in Hanoi providing services mainly for the population of the northern region in Vietnam. The exposure under study was proximity of cases and controls to the nearest reported PRRS outbreak in pigs (both in space and time). Since diagnostic services for bacterial meningitis including *S. suis* were limited at lower-level hospitals, suspected cases were often referred to NHTD for diagnosis and treatment. Therefore *S. suis*-infected cases diagnosed at NHTD can be considered as representative of all *S. suis* patients in the northern region. Sepsis was chosen as the control syndrome because *S. suis* patients and non*-S. suis* sepsis patients are similar in care-seeking behaviours and referral patterns, with non-*S. suis* sepsis assumed to be independent of the exposure of interest. Therefore, non-*S. suis* sepsis patients provide an estimate of background exposure rates of the study population [15]. The study was reviewed and approved by the Biomedical Research Ethics Review Committee at NHTD. Because human data were collected retrospectively from existing hospital records, no informed consent was obtained from the human cases and controls included in the study.

### Case definitions

A confirmed case of *S. suis* infection was defined as a patient who was admitted to NHTD in 2010, with *S. suis* infection confirmed either by standard bacterial culture or real-time polymerase chain reaction (PCR). In addition, the patient needed to have a residency address in the northern region of Vietnam, the geographical area which covered a total of 266 districts within 25 provinces as of 2010 (*Statistical Yearbook of Vietnam* 2010, see Supplementary Table S1)

A control patient was defined as a patient diagnosed with non-*S. suis* sepsis during admission at NHTD in 2010, and who also had a residency address in the northern region of Vietnam. Exclusion criteria for controls included: sign(s) of meningitis, laboratory evidence of *S. suis* infection, high suspicion of *S. suis* infection as determined by a doctor (despite being culture and PCR negative), and HIV infection.

### Data collection and variables

We identified *S. suis* case patients from the laboratory culture and PCR logbooks in the microbiological and molecular laboratory at NHTD, from which their medical records were traced back and retrieved. The majority of cases were filed under ICD-10 codes for meningitis due to bacteria or other/unspecified causes (G00, G03), while only a small proportion were categorized as unclassified sepsis (A41), unclassified viral encephalitis (A85) or viral infection of the central nervous system (A89). Sepsis control patients were retrieved using the ICD-10 code A41. Only those meeting inclusion and exclusion criteria for controls were included. For all included cases and controls, data captured included: sex, date of birth, outcome (survived/died), date of illness onset, date of admission, date of discharge/death, patient's address, occupation, history of eating high-risk pig dishes (raw pig blood or any other potentially undercooked pig products such as intestines, stomach or uterus), medical history, and clinical information.

Data on PRRS outbreaks in pigs was retrieved from the National Centre for Veterinary Diagnosis (NCVD) in Hanoi. NCVD serves as a national reference centre for surveillance and diagnosis of animal diseases; specimens from swine outbreaks are routinely submitted either directly to NCVD by farmers, private companies, district or provincial veterinary services, or are referred to NCVD through the network of regional animal health offices. We retrieved information on locations with PRRS-confirmed pig specimens in 2010, including date and location of specimen collection. These data were used as a proxy for geographical locations of PRRS outbreaks in pigs. A total of 1753 pig specimens from 35 provinces (mainly sera and offal) were tested for PRRS virus at NCVD in 2010 (Supplementary Table S2). PRRS positivity was recorded for 25·5% of the specimens across 57·1% of these provinces. Within the northern region, PRRS positivity was confirmed for 30·2% of the specimens from 18 provinces.

### Data analysis

We geo-coded addresses of human cases and controls, and the locations of PRRS outbreak farms using ArcGIS software (ESRI 2011, ArcGIS Desktop: Release 10, USA), and visualized these locations on maps using QGIS software (version 2.4.0, http://qgis.org). Descriptive analyses are presented in proportion [95% confidence interval (CI)] for categorical data, and mean (95% CI) and/or median (range) for continuous data.

We used both conventional logistic regression and space–time analysis to statistically examine the association between human *S. suis* cases and PRRS disease in pigs. For logistic regression, we classified all districts in the northern region into three corresponding categories: PRRS district (a district with a PRRS outbreak confirmed by NCVD), district adjacent to PRRS district (a district with no confirmed PRRS outbreaks but at least one outbreak confirmed in an adjacent district), and non-exposure district (no PRRS confirmation in the district or adjacent districts). We assigned exposure levels for each case and control patient by their residential location accordingly: residing in PRRS district, residing in a district adjacent to a PRRS district, and no exposure. ORs (95% CIs) were calculated to evaluate the significance of a factor in relation to the case and control status by univariable analysis. To examine the effect of exposure on disease status in the presence of existing potential confounders, we forced all significant factors (*P* ⩽ 0·10) into a model and removed step-by-step the least significant factors. We used Nagelkerke's *R*^2^ statistic to select the most parsimonious model with the highest explanatory power and smallest number of variables. Data collected from hospital notes on history of eating high-risk pig dishes are subject to potentially high levels of information bias since doctors might be more likely to ask patients with *S. suis* infection for this particular exposure compared to other patients. Therefore, we reported the results of the multivariable analyses with and without the inclusion of high-risk consumption history as a confounder.

For space–time analyses, we used bivariate *K* function to investigate the spatial interaction between human *S. suis* cases and controls, and space–time *K* function to test space–time interaction between human *S. suis* cases and PRRS outbreaks in pigs at the global scale (see Supplementary Methods). To detect space-time clusters of the *S. suis* cases at the local scale, we performed space–time scan statistics with SaTScan™ software (M. Kulldorff and Information Management Services Inc., SaTScan™ v. 9.2, www.satscan.org, 2013) using the Bernoulli model for case and control-type data [16]. Space–time clusters were identified by a moving circular window with varying diameters and a cylinder with varying heights of time (3-month interval). We analysed our dataset step-by step in three running sets to examine the possible clusters of *S. suis* human cases against controls as the background population with and without the input of PRRS pig outbreak location data as well as adjustment of sex, occupation, and eating history as potential confounding factors. Set 1: human *S. suis* cases and controls; set 2: human *S. suis* cases and controls, and PRRS pig outbreaks (PRRS outbreak locations were used as the centroid of the scanning window); set 3: human *S. suis* cases and controls were stratified into eight groups by sex, occupation and history of eating high-risk pig dishes (Table 1) (PRRS outbreak locations were used as the centroid of the scanning window).

**Table 1.** Groups of cases and controls included in running set 3 of the space–time SaTScan analysis

Group	Sex	Occupation	History of eating high-risk pig dishes
1	Male	Pig related/farmer	Yes
2	Male	Pig related/farmer	No
3	Male	Not pig related and not farmer	Yes
4	Male	Not pig related and not farmer	No
5	Female	Pig related/farmer	Yes
6	Female	Pig related/farmer	No
7	Female	Not pig related and not farmer	Yes
8	Female	Not pig related and not farmer	No

We defined parameters for the scanning windows following software guidance. The maximum cluster size was set at 50% of the population at risk for the spatial window and 50% of the study period for the temporal window. We used a Bernoulli model with likelihood ratios to evaluate statistical significance of this test, and *P* value was estimated from 9999 replications of Monte-Carlo simulations. The moving window with a maximum likelihood ratio was defined as the most likely cluster. Secondary clusters were only reported if no centroid was identified in the most likely cluster. Relative risk (RR) for each identified cluster was calculated as the ratio of the number of observed cases divided by the number of expected cases inside the cluster, and the number of observed cases divided by the number of expected cases outside the cluster.

## RESULTS

### Case-control analysis

**Fig. 1.**
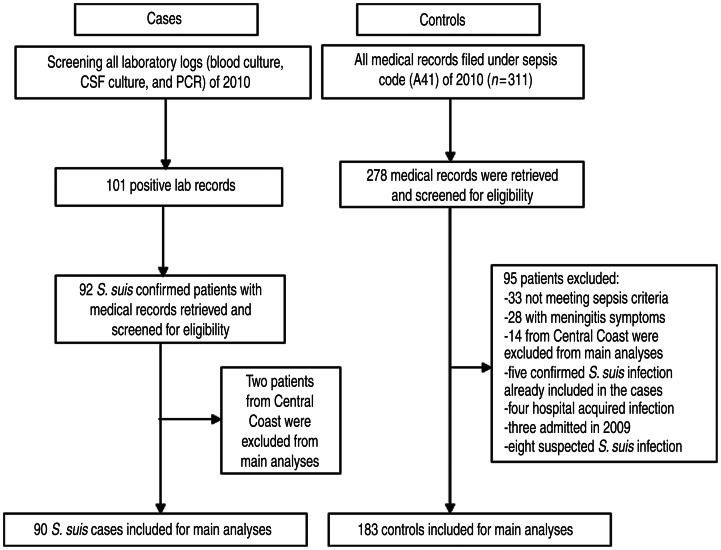
Identification and selection of cases and controls for the case-control study at the National Hospital for Tropical Diseases, Vietnam, 2010

A total of 90 *S. suis*-confirmed patients and 183 sepsis control patients were included in the main study analyses (Fig. 1). *S. suis* patients were similar to control sepsis patients in age, residential region, admitting departments and referral patterns (Table 2). However, compared to the controls, *S. suis* cases were more likely to be men, work in high-risk occupations (related to pigs/pig products or farmers), have a history of consuming high-risk pig products, and have a history of alcoholism. Regarding PRRS exposure, significantly more *S. suis* patients than controls (83·3%, 95% CI 75·6-91·0 *vs.* 62·3%, 95% CI 55·2-69·4, respectively) were from PRRS outbreak districts or neighbouring PRRS outbreak districts. Clinical information of these *S. suis* patients and control patients are available in Supplementary Table S3.

**Table 2.** Characteristics and behaviours of 90 human Streptococcus suis cases and 183 hospital controls included in the case-control study who were admitted to the National Hospital for Tropical Diseases in 2010

Variable	*S. suis* cases	Sepsis controls	*P* value*
Male sex	81 (90·0)	111 (60·7)	<0·001
Age, mean (95% CI)	48·5 (46·2–50·8)	50·6 (48·2–53·1)	0·273†
Residential region			0·506
North West	2 (2·2)	8 (4·4)	
North East	11 (12·2)	28 (15·3)	
Red River Delta	77 (85·6)	147 (80·3)	
PRRS exposure			0·002
Living in a PRRS district	27 (30·0)	44 (24·0)	
Living in a district adjacent to a PRRS district	48 (53·3)	70 (38·3)	
No exposure	15 (16·7)	69 (37·7)	
Occupation			<0·001
Pig-related	11 (12·2)	0 (0)	
Farmer	47 (52·2)	68 (37·2)	
Other	29 (32·2)	97 (53·0)	
No information	3 (3·3)	18 (9·8)	
History of eating high-risk pig dishes‡ prior to illness	27 (30·0)	1 (0·5)	<0·001
Alcoholism	28 (31·1)	39 (21·3)	0·077
Admitting department			0·219
Intensive care unit	48 (53·3)	108 (59·0)	
General infection	40 (44·4)	65 (35·5)	
Virology/Parasitology/Hepatitis	2 (2·2)	10 (5·5)	
Referred from other hospital/clinic	76 (84·4)	140 (76·5)	0·287

CI, Confidence interval; PRRS, porcine reproductive and respiratory syndrome.

Data are presented as *n* (%) unless otherwise specified.

* Difference was tested using Pearson's *χ*^2^ test unless otherwise specified.

† Difference in age means was checked using *t* test.

‡ Raw pig blood or potentially undercooked pig products such as intestines, stomach, uterus.

**Fig. 2.**
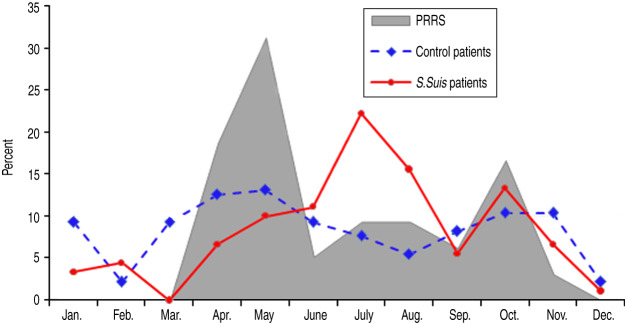
Distribution of porcine reproductive and respiratory syndrome (PRRS) positive pig specimens at NCVD, and *S. suis* human patients and control patients at the National Hospital for Tropical Diseases, Vietnam, 2010

Of 266 districts in the northern region of Vietnam, the number of PRRS districts, PRRS neighbouring districts and non-exposure districts was 49 (18·4%), 113 (42·5%) and 104 (39·1%), respectively. Figure 2 describes the temporal distribution of *S. suis* and control patients and PRRS-confirmed pig specimens. Data on pig specimens showed that PRRS outbreaks occurred between April and November, and the number of *S. suis* human cases admitted to NHTD was also higher in these months compared to other periods of the year. PRRS exposure was significantly associated with disease status by univariate analysis: *S. suis* patients were more likely to reside in a PRRS district (OR 2·82, 95% CI 1·35-5·89) and in a district adjacent to a PRRS district (OR 3·15, 95% CI 1·62-6·15) than control patients. However, living in a district adjacent to an area with PRRS outbreak activity but not in a PRRS district itself remained statistically significant in the final and most parsimonious models (Table 3). These models included PRRS exposure status, gender and occupation, with and without the inclusion of history of eating high-risk pig dishes. The adjusted ORs for living adjacent to a PRRS confirmed area were 2·60 (95% CI 1·27-5·34) and 2·19 (95% CI 1·01-4·75), respectively.

**Table 3.** Multivariable logistic regression analysis for 90 human Streptococcus suis cases vs. 183 hospital controls in 2010 in northern Vietnam

Parameter	OR (95% CI)	*P* value
**Model not including high-risk consumption history (Nagelkerke's *R*^2^ = 0·243)**		
PRRS exposure status (reference: no exposure)		
Living in a PRRS district	2·08 (0·94–4·62)	0·07
Living in a district adjacent to a PRRS district	2·60 (1·27–5·34)	0·009
Male sex (reference: female)	6·01 (2·77–13·05)	<0·001
Farmer/pig-related occupation (reference: non-farmer and non-pig occupation)	2·68 (1·52–4·74)	0·001
**Model including high-risk consumption history (Nagelkerke's *R*^2^ = 0·406)**		
PRRS exposure status (reference: no exposure)		
Living in a PRRS district	1·65 (0·69–3·93)	0·261
Living in a district adjacent to a PRRS district	2·19 (1·01–4·75)	0·048
Male sex (reference: female)	4·55 (2·02–10·20)	<0·001
Farmer/pig-related occupation (reference: non-farmer and non-pig occupation)	2·87 (1·53–5·38)	0·001
History of eating high-risk pig dishes prior to illness (reference: history not reported)	56·68 (7·35–436·95)	<0·001

OR, Odds ratio; CI, confidence interval; PRRS, porcine reproductive and respiratory syndrome.

### Space–time analysis

**Fig. 3.**
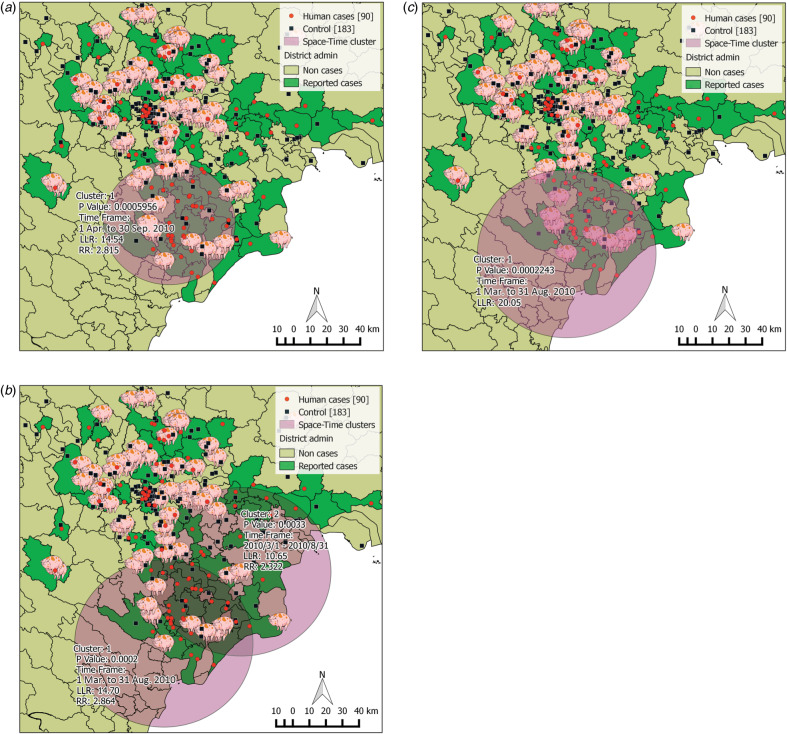
Clusters of *S. suis* cases detected in humans in three SaTScan running sets. (*a*) Set 1: only human cases and controls were used as input of the Bernoulli model. (*b*) Set 2: human cases and controls with porcine reproductive and respiratory syndrome (PRRS) locations as centroid of the moving space-time window. (*c*) Set 3: human cases and controls with eight groups of covariates by sex, occupation and history of eating high-risk pig dishes. Red dots represent human cases (90 cases); black square represent controls (183 controls); pig symbols represent locations confirmed with PRRS virus; purple circles represent the possible clusters constructed from SaTScan software. Cluster 1 is the most likely cluster, cluster 2 is the secondary cluster. For each cluster, *P* value, time-frame of the cluster detected, log likelihood ratio (LLR) and relative risk [RR; except panel (*c*)] are provided.

At the global scale, our bivariate *K*-function analysis suggested spatial clustering of *S. suis* human cases occurring at distances of 2–50 km (Supplementary Fig. S1). There was weak evidence of space–time interaction for both *S. suis* cases in human and PRRS outbreaks in pigs (Supplementary Fig. S2) at this global level. However, space–time analyses at the local level showed strong clusters of human *S. suis* cases occurring around locations where PRRS outbreaks were confirmed. Possible human *S. suis* clusters were found in all three running sets performed in SaTScan (Table 4). In running set 1, the most likely cluster was found within a radius of ~39·4 km from April to October (Fig. 3*a*). People who lived within the cluster had a higher risk of contracting *S. suis* infection than people living outside the cluster (RR 2·82). Using PRRS outbreaks for locating cluster centroids, the second running set found two likely *S. suis* clusters. The most likely cluster was diagnosed between March and August with a larger radius (53·6 km) (Fig. 3*b*) with a similar RR (2·86). In set 3, which included sex, occupation, and history of eating high-risk dishes as covariates, we only found one human *S. suis* cluster also between March and August (Fig. 3*c*). This cluster contained predominantly the four male patient groups with and without occupational exposure and history of high-risk consumption. The greatest risks were in men who worked in swine-related occupations and/or had a history of eating high-risk pig dishes (RRs from 3·78 to 6·0).

**Table 4.** Space–time clusters detected from SaTScan by three running sets

Set	Cluster*	Group†	Time frame (2010)	Cluster size (radius km)	No. obs.	No. exp.	LLR	RR	*P* value
1	1	n.a.	1 Apr. to 30 Sept.	39·38	24	10·3	14·54	2·82	0·0006
2	1	n.a.	1 Mar. to 31 Aug.	53·55	23	9·63	14·70	2·86	0·0002
	2	n.a.	1 Mar. to 31 Aug.	53·89	30	15·94	10·65	2·32	0·0033
3	1	1	1 Mar. to 31 Aug.	53·55	5	1·5	20·05	6·00	0·00022
		2			15	4·87		4·50	
		3			2	0·61		3·78	
		4			1	0·81		1·25	

No. obs, Number of observed cases; No. exp, number of expected cases; LLR, log likelihood ratio; RR, relative risk; n.a., not applicable.

* Cluster 1 is the most likely cluster.

† Groups are classified as in Table 1 for running set 3 only. Only four groups were included in the cluster identified from this running set.

## DISCUSSION

We examined the possible association between human cases of *S. suis* infection and PRRS outbreaks in pigs in the northern region of Vietnam using a case-control design. The coinciding increase in human *S. suis* cases and the number of PRRS outbreaks in pigs in 2010 has been reported previously [11], and we confirmed this association in this epidemiological investigation using logistic regression and space–time analysis. We showed that human *S. suis* infection tended to occur in areas with PRRS disease transmission in pigs, either in districts with confirmed PRRS outbreaks or in districts adjacent to at least one PRRS-confirmed outbreak. The spatial scan statistic has been useful in investigating clustering in case-control studies for malaria [17, 18], and sleeping sickness [19]. In our study, we were able to apply the confirmed space–time information on PRRS outbreaks in pigs to locate clusters of *S. suis* cases occurring in human populations. Our scanning circular windows around the PRRS outbreak locations identified a possible cluster of *S. suis* human infection between March and August, around the peak time of PRRS outbreak activity in northern Vietnam. The cluster contained predominantly men who had at least one exposure to pigs either through occupational contacts or eating practices.

The finding of increased risk for *S. suis* infection in those living in areas adjacent to PRRS transmission zones may be partially explained by the movement of infected pigs and pork products during outbreaks. Farmers frequently change marketing behaviours during outbreaks, with early sale of pre-market-weight pigs, or immediate sale of ill pigs as soon as symptoms appear [20, 21]. In our focus group discussions with community members in rural areas of northern Vietnam as part of a larger study on raw pig blood consumption [22], some farmers reported attempts to sell sick pigs even after a trade ban had been imposed on outbreak zones. Participating villagers from nearby non-outbreak areas also reported that traditional practices of consuming raw pig blood continued despite on-going swine outbreaks in neighbouring communes (V. T. L. Huong *et al.*, unpublished data). We included in the multivariable analyses two main, possibly confounding, factors; occupational exposure and eating high-risk pig dishes potentially contaminated with *S. suis* bacteria. This greatly improved the explanatory power of the logistic regression model as reflected in the higher Nagelkerke *R*^2^ value. However, since there might be a significant bias in recall of eating exposures between cases and controls, the results of both models with and without this factor are presented for comparison in this paper.

With the recurrent pattern of PRRS epizootics and its significant impact on the swine sector and farming communities [10], there is a high level of social awareness of PRRS (commonly known as ‘blue ear disease’). However, public awareness of associated human health risks of *S. suis* infection remains low [22]. By taking simple strategies such as safe practices of culling, slaughtering, and cooking, zoonotic transmission to humans can be relatively easily prevented. Efforts to reduce both PRRS and *S. suis* infection within pig populations through vaccination or other farming practices are needed and would benefit production output, farmers' livelihoods, as well as public health.

Since the first major waves of epizootic transmission in Vietnam in 2007, PRRS has become endemic throughout the country, and it remains one of the primary animal health concerns for the pig industry [23]. In the explosive fatal outbreaks of 2010, over 77 000 pigs were destroyed across the country, a much higher number compared to previous years [24]. Although PRRS virus was clearly the major causative agent of the observed morbidity and reproductive disorders of the 2007–2010 outbreaks, experimental studies using a Vietnamese PRRS virus isolate have failed to reproduce the severe clinical syndromes seen in the field [25], suggesting the involvement of secondary or concomitant infections that contribute to disease severity. *S. suis* was among the agents suspected of involvement, as were numerous other virulent swine pathogens such as classical swine fever virus, porcine circovirus type 2, porcine parvovirus, and *Mycoplasma hyopneumonia* [25–28]. PRRS might cause damage to swine pulmonary intravascular macrophages (an important factor in clearance of circulating bacteria), resulting in an increased susceptibility to bacterial infections such as *S. suis* [27]. Indeed, Hoa and colleagues showed an increased isolation rate of *S. suis* bacteria in sick pigs from PRRS-affected farms compared to healthy pigs in non-affected farms (11% *vs.* 0% of pigs affected by serotype 2) in Vietnam [11]. Nevertheless, lack of data on *S. suis* prevalence in diseased pigs in farms unaffected by PRRS in Vietnam precludes any conclusion on the link between PRRS in pigs and *S. suis* infection in humans.

The main limitation of this study is the ecological design and lack of more complete and detailed data on PRRS outbreak location, time and outbreak size and the exposure of cases and controls. In addition, differences in outbreak response activities and sample collection procedures could lead to under-reporting and under-estimation of the real prevalence and distribution of PRRS outbreaks. The ecological design of this study provides evidence of an association, but cannot conclude on causality. Exposure to PRRS for each individual patient was determined using district PRRS laboratory confirmations conducted at NCVD (group-level exposure data). Consequently, contextual effects within the shared environment (within-area variability) could not be accounted for [29]. Whether or not an individual living in a district with a PRRS outbreak or in a neighbouring district was exposed to PRRS-infected pigs or pig products may have been influenced by a number of individual characteristics. However, as informed by previous studies [5, 6], individual data on important variables including sex, occupation and history of eating high-risk pig products were also included to control for possible confounding. A prospective case-control study which investigates the exposures of cases and controls within a reasonable lag time period can increase the accuracy of the results. In addition, parallel investigations on *S. suis* prevalence in the PRRS-infected and non-infected pig herds in the targeted geographical region could provide more solid evidence of the association between human *S. suis* infection and PRRS outbreaks in pigs in combination with genotyping of *S. suis* isolates in pigs and humans, which could not be done in this study.

In conclusion, this study provides further evidence for an epidemiological association between *S. suis* infection in humans and PRRS disease in swine. Existing control strategies and regulatory activities such as trade bans and food inspection should be strengthened to prevent the movement, selling and consumption of sick pigs in outbreak zones and neighbouring areas. At the same time, programmes raising awareness are also needed to promote safe practices in the food chain production and safe consumption in the community.

## SUPPLEMENTARY MATERIAL

For supplementary material accompanying this paper visit http://doi.org/10.1017/S0950268815000990. click here to view supplementary material

## References

[1] Wertheim HF, *Streptococcus suis*: an emerging human pathogen. Clinical Infectious Diseases 2009; 48: 617–625. 19191650 10.1086/596763

[2] Goyette-Desjardins G, *Streptococcus suis*, an important pig pathogen and emerging zoonotic agent – an update on the worldwide distribution based on serotyping and sequence typing. Emerging Microbes & Infections. Published online: 18 June 2014. doi: 10.1038/emi.2014.45. PMC4078792 26038745

[3] Huong VT, Epidemiology, clinical manifestations, and outcomes of *Streptococcus suis* infection in humans. Emerging Infectious Diseases 2014; 20: 1105–1114. 24959701 10.3201/eid2007.131594 PMC4073838

[4] Mai NT, *Streptococcus suis* meningitis in adults in Vietnam. Clinical Infectious Diseases 2008; 46: 659–667. 19413493 10.1086/527385

[5] Wertheim HF, *Streptococcus suis*, an important cause of adult bacterial meningitis in northern Vietnam. PLoS ONE. Published online: 22 June 2009. doi:10.1371/journal.pone.0005973. PMC2696092 19543404

[6] Nghia HD, Risk factors of *Streptococcus suis* infection in Vietnam. A case-control study. PLoS ONE. Published online: 8 March 2011. doi:10.1371/journal.pone.0017604. PMC3050921 21408132

[7] Herold P, Breeding and supply chain systems incorporating local pig breeds for small-scale pig producers in Northwest Vietnam. Livestock Science 2010; 129: 63–72.

[8] Tisdell C. Trends in Vietnam's pork supply and structural features of its pig sector. The Open Area Studies Journal 2009; 2: 52–64.

[9] Dietze K, Porcine reproductive and respiratory syndrome (PRRS): virulence jumps and persistent circulation in Southeast Asia. In: *‘Focus on …’ series*. Rome: Food and Agriculture Organization of the United Nations 2011; 5: 8.

[10] Zhang H, Kono H. Economic impacts of Porcine Reproductive and Respiratory Syndrome (PRRS) outbreak in Vietnam pig production. Tropical Agricultural Research 2012; 23: 152–159.

[11] Hoa NT, *Streptococcus suis* and porcine reproductive and respiratory syndrome, Vietnam. Emerging Infectious Diseases 2013; 19: 331–333. 23343623 10.3201/eid1902.120470 PMC3559037

[12] Feng W, In utero infection by porcine reproductive and respiratory syndrome virus is sufficient to increase susceptibility of piglets to challenge by *Streptococcus suis* type II. Journal of Virology 2001; 75: 4889–4895. 11312360 10.1128/JVI.75.10.4889-4895.2001 PMC114243

[13] Galina L, Interaction between *Streptococcus suis* serotype 2 and porcine reproductive and respiratory syndrome virus in specific pathogen-free piglets. Veterinary Record 1994; 134: 60–64. 8135015 10.1136/vr.134.3.60

[14] Xu M, Secondary infection with *Streptococcus suis* serotype 7 increases the virulence of highly pathogenic porcine reproductive and respiratory syndrome virus in pigs. Virology Journal 2010; 7: 184. 20696031 10.1186/1743-422X-7-184 PMC2927530

[15] Grimes DA, Schulz KF. Compared to what? Finding controls for case-control studies. Lancet 2005; 365: 1429–1433. 15836892 10.1016/S0140-6736(05)66379-9

[16] Kulldorff M. A spatial scan statistic. Communication in Statistics – Theory and Methods 1997; 26: 1481–1496.

[17] Brooker S, Spatial clustering of malaria and associated risk factors during an epidemic in a highland area of western Kenya. Tropical Medicine and International Health 2004; 9: 757–766. 15228485 10.1111/j.1365-3156.2004.01272.x

[18] Coleman M, Using the SaTScan method to detect local malaria clusters for guiding malaria control programmes. Malaria Journal. Published online: 17 April 2009. doi:10.1186/1475-2875-8-68. PMC2679049 19374738

[19] Fèvre EM, The origins of a new *Trypanosoma brucei rhodesiense* sleeping sickness outbreak in eastern Uganda. Lancet 2001; 358: 625–628. 11530149 10.1016/s0140-6736(01)05778-6

[20] Tornimbene B, Knowledge, attitudes and practices of Cambodian swine producers in relation to porcine reproductive and respiratory syndrome (PRRS). Preventive Veterinary Medicine 2014; 116: 252–267. 24472214 10.1016/j.prevetmed.2013.12.009

[21] Nahar N, Pig illnesses and epidemics: a qualitative study on perceptions and practices of pig raisers in Bangladesh. Veterinaria Italiana 2012; 48: 157–165. 22718332

[22] Huong VTL, Raw pig blood consumption and potential risk for *Streptococcus suis* infection, Vietnam Emerging Infectious Diseases 2014; 20: 1895–1898. 25340391 10.3201/eid2011.140915 PMC4214319

[23] Nguyen T. PRRS Control in the Region. Compendium of Technical Items of the 28th Conference of the OIE Regional Commission for Asia, the Far East and OceaniaWorld Organisation for Animal Health, 2013 (http://www.oie.int/publications-and-documentation/compendium-of-technical-items/). Accessed 16 March 2015.

[24] OECD. Viet Nam. In: Livestock Diseases Prevention, Control and Compensation Schemes: Prevention, Control and Compensation Schemes. Paris: OECD Publishing, 2012, pp. 2189–2197.

[25] Metwally S, Pathogenicity and molecular characterization of emerging Porcine Reproductive and Respiratory Syndrome virus in Vietnam in 2007. Transboundary and Emerging Diseases 2010; 57: 315–329. 20629970 10.1111/j.1865-1682.2010.01152.x

[26] Rovira A, Experimental inoculation of conventional pigs with porcine reproductive and respiratory syndrome virus and porcine circovirus 2. Journal of Virology 2002; 76: 3232–3239. 11884547 10.1128/JVI.76.7.3232-3239.2002 PMC136035

[27] Thanawongnuwech R, Pathogenesis of porcine reproductive and respiratory syndrome virus-induced increase in susceptibility to *Streptococcus suis* infection. Veterinary Pathology Online 2000; 37: 143–152. 10.1354/vp.37-2-143 10714643

[28] Zhou L, Yang H. Porcine reproductive and respiratory syndrome in China. Virus Research 2010; 154: 31–37. 20659506 10.1016/j.virusres.2010.07.016

[29] Greenland S. Ecologic versus individual-level sources of bias in ecologic estimates of contextual health effects. International Journal of Epidemiology 2001; 30: 1343–1350. 11821344 10.1093/ije/30.6.1343

