# Supplementary material for: Temporal and spatial association of Streptococcus suis infection in humans and porcine reproductive and respiratory syndrome outbreaks in pigs in northern Vietnam
Source: Epidemiol Infect. 2015 May 22;144(1):35–44. doi: 10.1017/S0950268815000990 (PMC4697300; doi:10.1017/S0950268815000990)
Supplement: Supplementary file 1 [file S0950268815000990sup001.docx]

*Epidemiology and Infection*

Temporal and spatial association of *Streptococcus suis* infection in humans and Porcine Reproductive and Respiratory Syndrome outbreaks in pigs in Northern Vietnam

V. T. L. HUONG, L. V. THANH, V. D. PHU, D. T. TRINH, K. INUI, N. TUNG, N. T. K. OANH, N. V. TRUNG, N. T. HOA, J. E. BRYANT, P. W. HORBY, N. V. KINH, H. F. L. WERTHEIM

**Supplementary Material**

**Methods**

*Global space-time analyses*

We used bivariate K function to investigate spatial interaction of *S. suis* cases compared to controls which account for inhomogeneous population [[1](#_ENREF_1)] at global scale, namely entire study area. The bivariate K function estimate is defined as:

$K_{ij}\left( d \right)=\lambda_{j}^{-1}\left[ \begin{aligned} expected number of type j events within distance d of a randomly selected \\ type i event \end{aligned} \right]$(E.q.1)

$\lambda_{j}$is the number of events j expected per unit area. In this case, event *j* is the controls and event *i* is the cases. Results of K function were then transformed to a linear presentation for intuitive interpretation by L function, defined as $L_{ij}\left( d \right)= \sqrt{\frac{K(d)}{\pi}}-d$ (E.q.2)

In addition, space-time K function (extended from K function [[2](#_ENREF_2), [3](#_ENREF_3)]) was also applied to identify space-time interaction among *S. suis* cases and PRRS disease in pigs. By incorporating temporal effect, the space-time permutation of the cases is defined as:

$K\left( d,t \right)= \lambda_{d}^{-1}\lambda_{t}^{-1}\left[ \begin{aligned} expected number of cases within distance d and \\ time t of a randomly selected case \end{aligned} \right]$ (E.q.3)

$\lambda_{d}$is the number of cases in space per unit area and $\lambda_{t}$ is the number of cases in time per unit area. The excess risk $\hat{D}o(d,t)$ due to space-time interaction was used to diagnose results. $\hat{D}o(d,t)$is a transformation of the difference $\hat{D}\left( d,t \right)$between space-time permutation and product of spatial K function and temporal K function.

$\hat{D}\left( d,t \right)=K\left( d,t \right)-K\left( d \right)K(t)$ (E.q.4)

$\hat{D}o\left( d,t \right)=\frac{\hat{D}\left( d,t \right)}{K\left( d \right)K(t)}$ (E.q.5)

Monte-Carlo (MC) simulation running in 999 iterations was used for statistical significance test of both K functions. All global tests were implemented in R program with spatstat [[4](#_ENREF_4)] and splancs [[5](#_ENREF_5)] package.

**Supplementary Table S1.** *List of northern provinces of Vietnam as of 2010 (Statistical Yearbook of Vietnam 2010,* [*http://www.gso.gov.vn*](http://www.gso.gov.vn) *)*

| **Red River Delta** | **North East** | **North West** |
| --- | --- | --- |
| Thai Binh | Thai Nguyen | Hoa Binh |
| Hung Yen | Bac Giang | Son La |
| Bac Ninh | Cao Bang | Dien Bien |
| Hai Duong | Quang Ninh | Lai Chau |
| Nam Dinh | Bac Kan |  |
| Ha Nam | Lao Cai |  |
| Vinh Phuc | Tuyen Quang |  |
| Ninh Binh | Yen Bai |  |
| Hanoi | Phu Tho |  |
| Hai Phong | Lang Son |  |
|  | Ha Giang |  |

Note: Provinces in the red-colored font had PRRS confirmation in pigs as recorded at NVCD in 2010.

**Supplementary Table S2.** *Pig specimens sent to NCVD and proportion of PRRS positive specimens in 2010*

|  |  | No. specimens | No. batches | No. provinces with specimens |
| --- | --- | --- | --- | --- |
| Nation-wide (63 provinces) | Total | 1753 | 246 | 35 |
|  | Positive | 447 | 121 | 20 |
|  | % positive | 25,5% | 49,2% | 57,1% |
| Northern (25 provinces) | Total | 1284 | 227 | 21 |
|  | Positive | 388 | 118 | 18 |
|  | % positive | 30,2% | 52,0% | 85,7% |

**Supplementary Table S3.** *Clinical information of 90 S. suis patients and 183 control patients admitted to NHTD in 2010*

| Variable^*^ | *S. suis* patients | Control patients | p value^†^ |
| --- | --- | --- | --- |
| Duration from onset to admission (d) (median (range)) | 2 (33) | 5 (61) | <0.001^§^ |
| Antibiotic use at admission (n(%)) | 34 (37.8) | 51 (27.9) | <0.001 |
| Neck stiffness | 78 (86.7) | - |  |
| Difficult hearing | 40 (44.4) | 2 (1.1)^¶^ | <0.001 |
| Fever | 90 (100) | 180 (98.4) |  |
| Skin rash (any) | 20 (22.2) | 35 (19.1) |  |
| Purpura fulminans | 15 (16.7) | 1 (0.5) | <0.001 |
| Hospitalization duration (d) (median (range)) | 15 (51) | 9 (99) | <0.001^§^ |
| Complications |  |  |  |
| Acute renal failure | 14 (15.9) | 52 (28.4) | 0.025 |
| Acute liver failure | 5 (5.7) | 16 (8.7) |  |
| Endocarditis |  | 6 (3.3) | 0.09 |
| ARDS | 5 (5.7) | 23 (12.6) | 0.08 |
| Coagulopathy | 4 (4,5) | 5 (2.7) |  |
| Septic shock | 5 (5.7) | 54 (29.5) | <0.001 |
| Treatment |  |  |  |
| Corticosteroids | 66 (75.0) | 21 (11.5) | <0.001 |
| Oxygen supplementation | 20 (22.7) | 104 (56.8) | <0.001 |
| Inotropes / vasopressors | 9 (10.2) | 61 (33.3) | <0.001 |
| Mechanical ventilation | 11 (12.5) | 46 (25.1) | 0.017 |
| Continuous Veno-Venous Hemofiltration | 1 (1.1) | 6 (3.3) |  |
| Blood Transfusion | 9 (10.2) | 68 (37.2) | <0.001 |
| Outcome |  |  | <0.001 |
| Death | 6 (6.6) | 47 (25.6) |  |
| Recovery with sequelae: | 26 (28.9) | 8 (4.4) |  |
| *Hearing loss* | *15 (16.7) )* | - |  |
| *Tinnitus* | *8 (8.9)* | - |  |

*Data are presented in n (%) or otherwise specified. † p-value is reported if it was <0.10. Difference was tested using Pearson Chi Square Test or otherwise specified.

^§^Mann-Whitney U Test. ^¶^One had a history of deafness since birth and one had had deafness for 30 years.

**Supplementary Figure S1.** *Diagnostic plot of bivariate K function of S. suis cases and controls. Red line is the estimated values of observed data. Blue line is spatial independence. Red line above blue dash line indicates attraction of S.suis cases and controls. Gray shade is a pointwise envelope, the null hypothesis will be rejected if observed value falls outside the envelope at a certain estimated distance.*


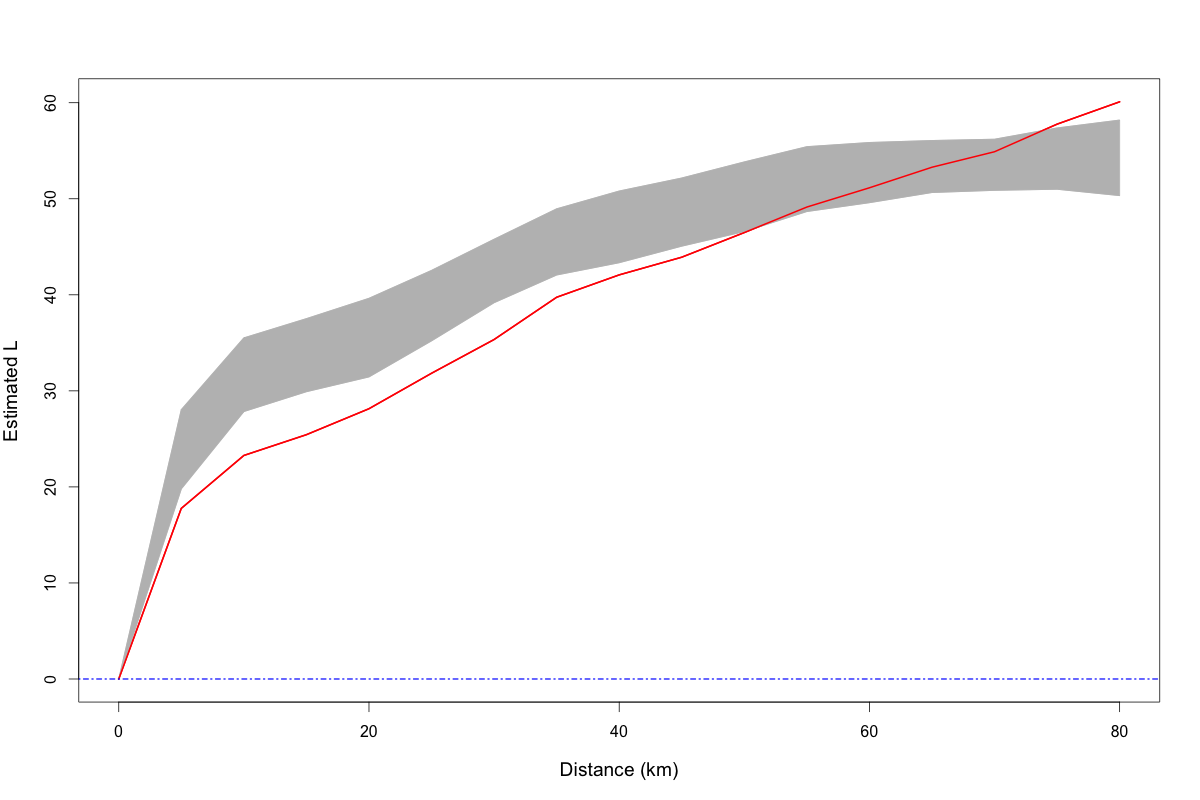


**Supplementary Figure S2.** *Diagnostic plot of space-time test for PRRS and S.suis cases. a) PRRS, perspective plot of* $\hat{D}o(d,t)$*; b) PRRS, scatter plot of* $R\left( d,t \right)$*against* $\hat{K}(d)\hat{K}(t)$*; c) S.suis cases, perspective plot of* $\hat{D}o(d,t)$*; d) S.suis cases, scatter plot of* $R\left( d,t \right)$*against* $\hat{K}(d)\hat{K}(t)$*.*

*
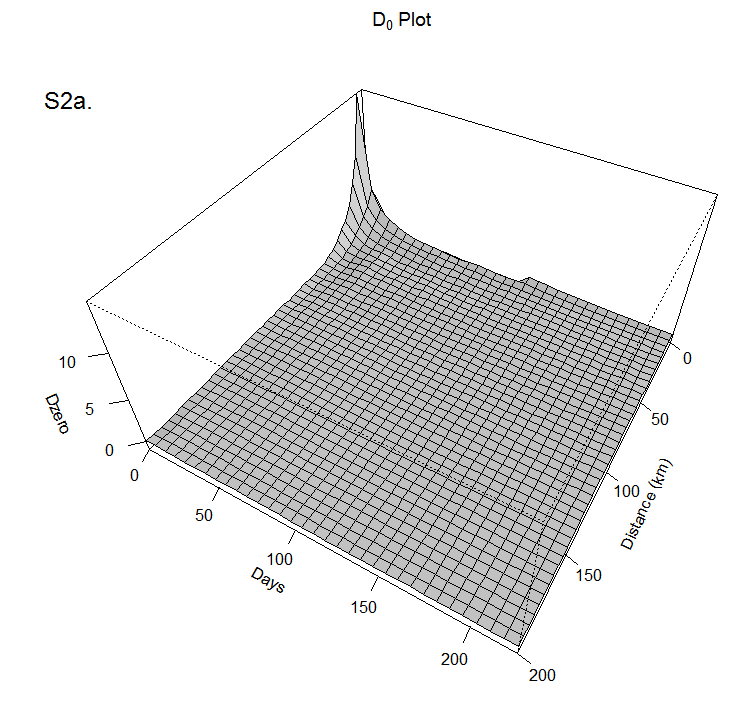
*

***
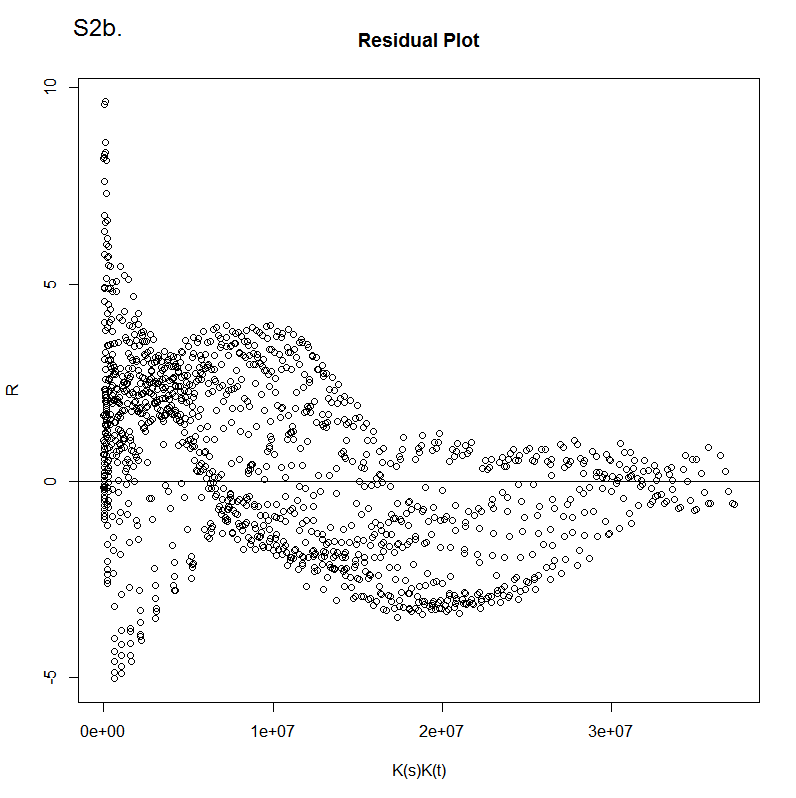
***

***
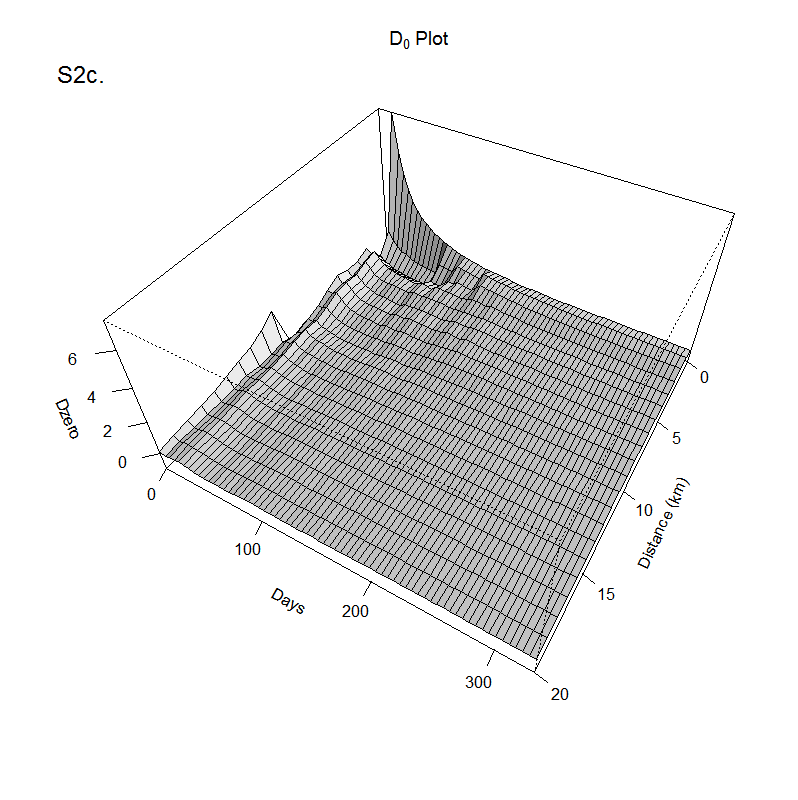
***


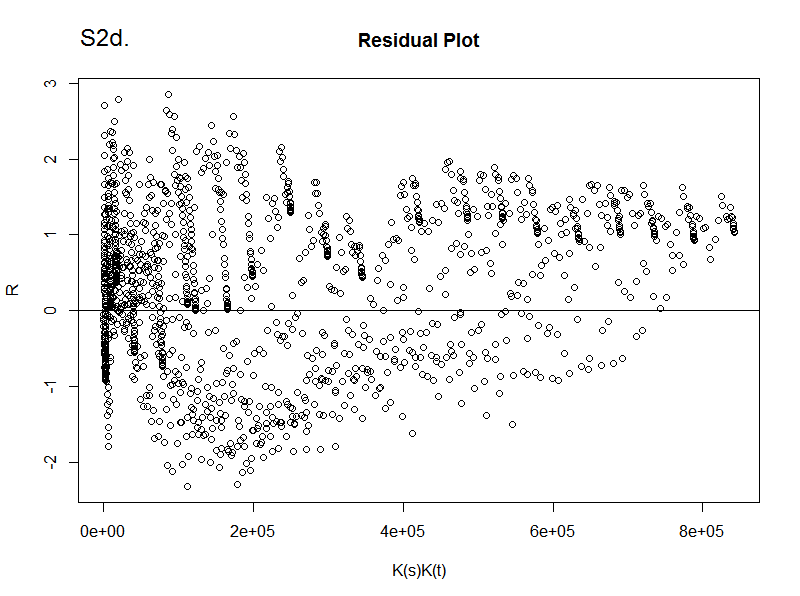


Summary on the Supplementary Figures:

Figure S1 shows significant evidence of interaction between *S. suis* cases and controls with likely attraction at distances from 2 km to 55 km. Weak evidence of space-time permutation was found in both *S. suis* cases in humans and PRRS incidence in pigs (Figure S2a,b,c,d). The perspective plot for PRRS (Figure S2a) indicates an excess risk existing in a distance of 35 km in space and 40 days in time but the evidence was not strong (residuals distributed on both sides of the horizontal axis in Figure S2b, upper tail p-value 0.66). Likewise, for the *S. suis* cases (Figure S2c), the excess risk was found in a distance of 5 km in space and 90 days in time (residuals distributed on both sides of the horizonal axis shown on Figure S2d, upper tail p-value 0.77).

**Supplementary References**

1. Diggle, P. and A. Chetwynd, *Second-Order Analysis of Spatial Clustering Inhomogeneous Populations.* Biometrics, 1991. **47**(3): p. 1155–1163.

2. Diggle, P., et al., *Second-order analysis of space-time clustering.* Stat Methods Med Res, 1995. **4**(2): p. 124–136.

3. Gatrell, A.C., et al., *Point Spatial application pattern analysis geographical epidemiology.* Trans Inst Br Geogr, 1996. **21**(1): p. 256–174.

4. Baddeley, A. and R. Turner, *spatstat: An R Package for Analyzing Spatial Point Patterns.* J Stat Softw, 2005. **12**(6): p. 1-42.

5. Rowlingson, B.S. and P. Diggle. *splancs: Spatial and Space-Time Point Pattern Analysis*. 2013 29Sep2014]; Available from: <http://cran.at.r-project.org/web/packages/splancs/index.html>.
